# Proceedings of the Maryland and District of Columbia Dental Society

**Published:** 1879-11

**Authors:** 


### ARTICLE III.

[Continued from last Number.]

### *Proceedings of the Maryland and District of Columbia Dental Society.—Annual Meeting.*

BALTIMORE, Wednesday, Sept. 3rd, 1879.

The President called the meeting to order at 11 A. M. The minutes of the previous meeting were read and adopted.

Dr. R. B. Winder nominated Dr. Samuel Johnson, of Baltimore, who was duly elected a member of the Association.

Miscellaneous business being called, Dr. Winder took the floor and said:—

*Mr. President.*—There has been a desire this morning to urge me to make some remarks, and call the attention of the Association to the present "three-fourths vote" system of the selection of members. I think it would be advisable to appoint a committee to report upon each candidate for membership, so that we can be more careful to know what we are doing. There has been some complaint that our present system is too loose; that men are allowed to get in here who are not fit to be members of this Association. By some it has been advised that a standing committee be appointed to investigate and report the fitness or unfitness of each candidate. I desire also to call the attention of the Association to another matter. Other States have been making a movement towards obtaining appropriate legislation for governing the practice of dentistry, and it is time we were making such a movement for our territory. If that subject is to come up all the gentlemen present can determine what is best to be done. Many States have taken actions in the past year. Of course no legislation could interfere with those already in practice.

In some States they require that a practitioner shall either pass an examination before a Board of Censors appointed by the State, or that he shall have graduated at some dental school. This is not in accordance with my own view. A diploma does not show that its holder is qualified to meet whatever demands may be made upon him in his profession. My own views have always been that we should go to the bottom of the thing, and require of the practitioner, first a diploma, and then an examination. At the same time we must remember that in dealing with these things we are running ahead of the medical profession itself, because States generally do not require a diploma. If the masses are so careless where so much is at stake, they may consider that we are running ahead of this old profession. I should like to hear from our medical men. I should like to have them take the same step we are taking. It would be even better for the medical profession to take the advance, leaving ours, the junior profession to follow.

Dr. Volck in a few remarks confirmed the opinion of Prof. Winder.

Dr. Scott.—I regard this as a most important subject. It is but too true that the standard of graduation is deplorably low. It is but too true that diplomas may be had too readily. It has often been charged that they can be bought in the public market in certain of our cities. Hence I join with my friend, Prof. Winder, in any scheme which will promote the advancement of our professions. And I notice in the newspapers that in Virginia an effort was made to establish a board of examiners. A few years ago the same thing was attempted. They wanted to require a diploma and then an examination. That is what should be done. The dental profession may have the honor of inaugurating a system which tends somewhat to avert the evil, and that is to prevent those who lecture from examining the students for graduation. In conversation with Dr. Winder, I find he favors it very highly, and I thought something of that sort would tend to elevate the standard. The medical profession have done nothing, and it appears to me it is getting from bad to worse. Fees even lower; some of them I understand, twenty five dollars for the course; two sessions to graduate, one in the summer and one in the winter. When we form medical associations among ourselves, when we form dental associations, we can regulate the membership for ourselves so far as science is to be a basis and so far as individual qualification is to be a standard. Membership will be sought for. I do not agree with Dr. Winder that we should wait for the medical men. I want him to take the lead. His profession has taken the lead in one thing, now take it in another. Have the candidate not only a man in character, but demand that he shall be fit to pass an examination.

Prof. Winder offered a motion to the effect that the two subjects just discussed be passed for the present, and called up at the discretion of the President sometime during the day. The motion was carried.

On motion of Dr. Hunt the organization of the sections was made the special order at the evening session.

The amendment to the constitution abolishing the office of Corresponding Secretary, and transferring his duties to the Recording Secretary, proposed last year, was then adopted.

Dr. Wm. B. Ulrick, of Chester, Pa., was then on motion of Dr. Hunt, elected to honorary membership.

Dr. Ulrick being invited, took the floor and said:

*Mr. President*: Dr. Caldwell embarrasses me. I came here at his solicitation, not to speak, but to see the scope that an organization of this kind would take. I have been exceedingly interested—interested upon a subject that I have long had at heart. I am not ignorant of the fact that the great progress of dentistry has kept astride with the progress of medicine. I recognize myself the close intimacy with my own profession. I not only look upon it as collateral with that profession, but as more intimately connected with it than many of the votaries of my own profession recognize, or are prepared to recognize. Now I did not come here to talk. I did not come here to make any suggestions that might guide you to a more elevated position. I came here to listen; but when the subject of an elevated standard of my own profession is up for discussion, it is not very troublesome to enlist me in it. The gentleman who sits before me made suggestions it seems to me in the proper direction. I do not believe that legislation can have much influence. I believe the matter rests in the hands of the members not only of the dental profession but of the medical profession. Elevate our own standard, but how shall we best do it? By organizations of this kind; by meeting and throwing together our combined information and elevating the standard. Making these organizations, or societies, worth seeking. The public soon recognizes the fact that the man who is willing to measure his ability is the man to tie to. And those who keep outside of the organizations, will be the first to be frowned upon. So you, gentlemen of the Dental Association of the State of Maryland and District of Columbia, will bring about a more elevated course of instruction in your profession. I make these random remarks; I have no conceited views upon the subject, but I feel that your presence here to-day, and the discussions that are to take place here must advance you, must bring you to the notice of the general public, that are growing more and more discerning from our public schools. With my very best wishes for the Dental Association of Maryland and District of Columbia, and its individual members, I take my seat.

Reports of business committees being the next order, Dr. H. B. Noble, chairman of scholarship committee, offered the following report.

The committee appointed to report on scholarship, beg leave to offer the following as their report:

That in their opinion, it would be better that a competitive examination should be held, the one who is best qualified to receive the benefit.

H. B. Noble, *Chairman.*
M. W. Foster.

The difficulty seeming to be that there were only two members of the committee in attendance on the meeting, on motion the committee was filled by the appointment of a third temporary member, and the report was recommitted to the committee, with instructions that they should make the selection in such way as in their discretion was best.

The President appointed Dr. Donaldson to fill the vacancy temporarily.

Dr. Noble made the following report of the Treasury.

BALTIMORE, Sept. 3rd, 1879.

*Treasurer Reports:*

| | |
|---|---|
| Deficit at last report, - - - - - - | $ 7.91 |
| Paid to Reporter, Dr. H. M. Schooley, - - | 25.00 |
| | $32.91 |
| Received from Dues and Assessments, - | $50.50 |
| | 32.91 |
| Balance in Treasurer's hands, - | $17.59 |

Subject to printing bill of $118.00, and other expenses of the present session, leaving the Society over one hundred dollars in debt.

DR. HUNT.—I move that the report just heard be referred to the executive committee to report ways and means to meet the deficiency. Carried.

On motion of Dr. Winder, Dr. Scott's paper was passed.

On motion, section four was passed.

Section second being called, Dr. W. H. Atkinson, of New York, read the following paper:

*Report of Section 2,—Histology and Microscopy.*

W. H. ATKINSON, M. D. *Chairman.*

In accordance with instructions, I wrote the members of my section early in the season. Three replied, and four failed to notice the matter at all. Having been overborne with a multiplicity of responsibilities of a like character to our professional bodies, I have had little time or opportunity to prepare such a paper as I should desire to present to this body as a truly scientific statement of just what I understand Histology and Microscopy to be. In the absence of assurance of any help in the way of partial or supplemental papers upon the subjects committed to our section, out of which to collate a condensed report embodying the work of the several members of the section, I have determined to offer the following lay-out as a sort of basal or foundational postulate from which to proceed in building the real body of histological knowledge only knowable by correct application of Microscopical love.

I have been for some time past a student of Universology under the personal instruction of Stephen Pearl Andrews, the discoverer and formulator of that new and wonderful science; and he does me the honor to say that I aid him by my greater familiarity with the details of several specialties, and adds that a certain similarity of mental appetite and pursuits creates an unusual degree of intellectual sympathy between us. I propose on this occasion, to take a very simple first-step towards initiating you into an understanding of how universological discovery is about to affect, and perhaps, entirely recast Histology, or Microscopical Anatomy, which as you are aware is at the bottom of our branch of science, which is dental surgery. Our method of arriving at results is this. Mr. Andrews, taking the lead, makes a provisional application of universological principles, in a sort of *a priori* way to the histological special field of knowledge, upon the basis of the prevalent views upon that subject. To this first projection I then add my criticism and my contribution from my own observations, and from my greater familiarity with the discoveries of Carl Heitzman, Louis Elsberg, F. C. W. Bœdecker, (here justly named,) and others, the most recent and competent investigators in this special fi ld. When then we have fully discussed a proposed conclusion, and arrived at unanimity as between us two, we consider the matter sufficiently settled to be presented for the consideration of the learned world. But the learned world must be slow in condemning or fully approving, inasmuch as all that can be exhibited at once is but a brick or stone, to be wrought into place in the structure of an immense edifice. What I am about to show is then, merely a specimen brick.

After certain preliminary stages, the real beginning point of embryonal development, and so of the proper science of Embryology itself, is the differentiation of the embryonal pellicle into layers; and, as the extant authorities express the fact, into an external and an internal layer, recognizing a twofold division merely. From the outer layer or ectoderm the *dorsal plates* are said to arise which, closing in backwise around the locality of the spinal cord and brain, constitute that which Richard Owen named the "Neural" or "Nerve Cavity;" and from the internal layer or endoderm the *abdominal plates* arise, and closing in frontwise, there is formed what he named the "Hæmal" or "Blood Cavity."

Observe, in passing, that there is necessarily, or by the natural admixture of parts, a portion of blood in the nerve cavity, a portion of nerve substance in the blood cavity, and a portion of other matters neither nerve nor blood in both; and that, so, all such physiologically local discriminations are to be taken as limited by the universological principles of NERVE PREPONDERANCE OR OVER-LAPPING and of PREDOMINANCE AND SUBDOMINANCE, by which universology carefully guards against misapprehension.

Following this idea of a twofold distribution of the materials, embryologists have proceeded to designate the mass-growths of the ectoderm and the endoderm; and to give a clear understanding of how they view this very important matter. I quote the following passages from the article in Johnson's Cyclopædia, on Embryology, by J. C. Dalton, one of the best and most recent *published* authorities. "The dorsal and abdominal plates as they grow thicker and more condensed, begin to show, in their substance, the distinction of the various tissues. The external integument

(the skin,) the tissue of the voluntary muscles, the cartilages and bones, the organs of special sense, the nerves of sensation and voluntary motion, and the white and gray matter of the brain and spinal cord, are thus formed in the substance of the growing material. All the organs and tissues just enumerated, notwithstanding their different functions, are closely related to each other in one respect; that is they are destined to bring the animal body into relation with the external world by means of sensation, consciousness, volition, voluntary movement, and the mechanical reception and expulsion of nutritious or effete materials. They are accordingly known as the organs of animal life, and they are all formed from the original cells of the *external layer* of the blastodermic membrane.

"There is also, however, an internal layer of the blastodermic membrane; and from this layer are formed the alimentary canal and its glandular appendages, or the organs in which digestion, absorption, and secretion are to be carried on, and in which the muscular actions are involuntary and unconscious. They may, therefore, be regarded as 'the organs of vegetative life.'" * * * * "These are the general features of the development of the embryo in all vertebrated animals."

Without in any manner interfering with this twofold distribution; leaving it intact for all it is worth; and acknowledging its validity and importance as a preliminary and initiative form of discrimination, universology points out the fact that it is a somewhat jumbled and confused account of the matters in hand, and propounds the further—on proposition that it is not the two-fold, but the *three-fold* basis of discrimination which puts the keystone in the royal arch of construction, and furnishes the satisfactory and ultimate system.

The jumble in the two-fold arrangement appears in the fact that the outermost integument, the innermost nerve ganglia, and the intermediate bone system are thrown together in one class, while interstitial matters make the other class; this appears by the fact that the dead, stone-like bony system is classed among the more highly vital "organs of animal life," etc. The three-fold arrangement which universology introduces, overlies, underlies, and embraces the two-fold arrangement, bringing order out of chaos. This is the specimen brick which I have referred to, and which I will now proceed to present. According to this conception the embryonal sheet separates really not merely into two, but into three layers, the middle one of which is *Neutral*, and as a mere *residuum* and least highly organized part constitutes the *really central portion* of the new being, considered as a fabric, consisting of cartilage, tendon, and bone—in a word the Osseous System. This intervenes with its own main structural embodiment, between the neural and the hæmal cavities, the so-called *centra* of the spinal column being its centre, whence it sends out spines backward and ribs (also spines) forward, closing around and enclosing the neural and hæmal cavities, respectively.

In the course of development, the neural cavity is so shrunken in size, throughout the whole length of the body except in the head, that the presiding energy seems to vacillate between preserving the type idea of two cavities, and on the other hand, treating the whole as one cavity. It is in accordance with the theory of two cavities that the spinal cord is kept in its distinct canal throughout; but in all other respects, the whole barrel of the trunk is treated as a single cavity having appropriate contents and walls. The skin, therefore, envelopes the whole as one; while the bowel as *in*-skin occurs only in the one big cavity, along with the heart and the sympathetic nerve system; and finally, the muscle is distributed partly to the exterior and partly to the interior of this cavity.

It will now appear that we are authorized to place the bony system by itself, as the analogue of the MINERAL KINGDOM and to call its parts the organs of Mineral life in the same analogical sense as that in which physiologists have so habitually spoken of the organs of Vegetative life and the organs of Animal life. In other words, the middle neutral plate developing into the osseous system reproduces and represents the Mineral Kingdom; the abdominal cavity with its digestory and circulatory apparatuses reproduces and represents the Vegetable Kingdom; and the neural cavity and its contents, backed by the general integuments (the exterior muscles and the skin,) reproduces and represents the Animal Kingdom,—all within the constitution and organization of the individual animal or human body; thus in a sense rounding out, and, as it were, merely initiating the grand universal system of analogy between the outer cosmical and the inner or individual world.,

The terms Masculoidium or Nocturnium, applied to the neural cavity and contents, and the terms Femenoidium or Diurnium applied to the abdominal cavity and contents, glance merely at ulterior universological discriminations to which we cannot give our attention now. The interior bony system of the vertebrates hitherto was called neutrum, but must now be discriminated as endo-neutrum or the endoskeleton, reserving the terms exo-neutrum and exo-skeleton for the outer skin and its armature.

On motion of Dr. Foster, the Society took a recess until half-past two.

After recess the President announced the paper on histology and microscopy open for discussion.

Dr. Winder.—Mr. President, we certainly should not let this paper pass without something being said about it. I have never been a histologist, but I recognize the fact that if you will take these diagrams and study them carefully it will give all a very clear conception of embryonal growth. He has given there a representation of all parts and the manner in which they are developed. It is a paper that deserves to go on record, and these diagrams are within the comprehension of any man who will look at them and think. They show there the typical form of man. You will find this same thing in each of the two sections no matter which way you cut. This is very beautifully expressed in one of Huxley's works. It is the typical form of vertebrate life. When I lectured at the Maryland College on physiology, my very first lecture was commenced right there on the typical form of vertebrate life.

The second figure is original with Dr. Atkinson I presume. It is just an extension of the other, and carries the whole facts right to the mind of anyone desiring information. It is plain, simple, and intelligible.

Dr. Atkinson offered some remarks with illustrations on the black-board.

On motion the subject of Histology and Microscopy was passed.

Dr. Hunt, Chairman of Section sixth,—Metallurgy and Chemistry of Metals—read the following paper:

The section having charge of "Metallurgy and Chemistry of the Metals" respectfully report, that owing to a combination of circumstances during the past year, the section has been able to do little more than examine the field of its labor and map out its work for the future.

It is the desire of the Section to pursue its researches systematically and carefully, and to present to the Association only the processes and results of such careful and thorough investigation.

To mark out more definitely the work to be done, it is intended to give to the term "Metallurgy" its most extended signification, so as to embrace the workings and behavior of metals, both singly and in all combinations with each other, so far as they are applied or are proposed to be applied to the practice of our profession. This refers only to their metallic state, as in alloys, amalgams, etc. The study of the "chemistry of the metals" will consider their relations and behaviour when their states have undergone a chemical change, as in chlorides, oxides, etc.

Most of the work hitherto done in the way of using metals for the purposes of our profession has been empirical rather than scientific. But it is understood that the investigation of this subject is now being conducted in a scientific and practical manner by the "New Departure Corps." Our section will doubtless receive valuable assistance from that quarter, and will cheerfully co-operate with them and all others working in that direction.

In pursuing our work we will necessarily trench somewhat on the domain of "Operative Dentistry," but in doing so it will be our endeavor only to act as an auxiliary to the section on that subject, and we will most probably communicate to its Chairman from time to time any conclusions or results at which we may have arrived, coming naturally within its province.

The section desires to present at this time to the Association three conclusions:

1.—That pure gold introduced into a tooth as a filling is inert and *of itself* exercises no electric or galvanic action on the tooth.

2.—That while amalgam fillings conform to the general shape of the cavities, the surfaces of the fillings do not fit closely to the surfaces of the cavities, but are rough and uneven or indented, the indented or depressed portions not coming in contact with the dentine, thus being liable to leak, and in fact a very large number do leak.

3.—That plastic fillings into the composition of which chlorine enters, cannot be relied upon as permanent; first, because of the great affinity of chlorine for alkalies. second, because the other constituents of the filling have greater affinity for other acidulous agents than for chlorine, —these affinities bringing about a disintegration of the filling.

On motion of Prof. Winder, the report of Sec. VII was passed for the time.

Dr. Winder.—As Chairman of Executive Committee, I report that we find that an additional assessment of two dollars from each member will re.ieve us of our financial embarrassment, provided that the American Journal of Dental Science will print our proceedings instead of printing them ourselves. Report adopted.

Adjourned until 8 P. M.

